# Supplementary material for: Development of a Microwell System for Reproducible Formation of Homogeneous Cell Spheroids
Source: Pharmaceutics. 2025 Dec 31;18(1):56. doi: 10.3390/pharmaceutics18010056 (PMC12844868; doi:10.3390/pharmaceutics18010056)
Supplement: Supplementary file 1 [file pharmaceutics-18-00056-s001.zip › pharmaceutics-4037138-supplementary.pdf]

Development of a Microwell System for Reproducible Formation of Homogeneous Cell Spheroids

Miguel A. Reina Mahecha [1], Ginevra Mariani [1], Pauline E. M. van Schaik [2], Paulien Schaafsma [1], Theo G. van Kooten [1], Prashant K. Sharma [1] and Inge S. Zuhorn [1,*]

Supplementary information

Code 1*. Macro code to identify the radii of the EB or cell aggregates on FIJI:*

```
#@ File (label = "Input directory", style = "directory") input
#@ File (label = "Output directory", style = "directory") output.
#@ String (label = "File suffix", value = ".tif") suffix

run("Set Scale...", "distance=912.7437 known=1000 unit=um global");
processMaxiFolder(input);
/*
Made by Alejandro Reina Mahecha 14/11/22, to use this code, please reference the paper
 */
// function to scan a folder with multiple folders e.g. 24h, 48h and 72h
function processMaxiFolder(input) {
	list = getFileList(input);
	for (i = 0; i < list.length; i++) {
			processFolder("" + input + "/" + list[i], list[i]);
			waitForUser("next folder");
	}
}

//Function to find the folder that contains the images, e.g. PDMS with AARS, PDMS without AARS
function processFolder(input, folder) {
	list = getFileList(input);
	for (j = 0; j < list.length; j++) {
			processFile(input, "" + folder + list[j]);
	}
}

// Function to process each image
function processFile(input, file) {
	open(file); // opens file
	run("8-bit"); // transform the RGB image into 8 bit
	setTool("oval"); // tool that the user will use to select the sphere
	waitForUser("select the sphere"); // user selects the sphere
	run("Duplicate...", " "); // The program makes a new image only with the selected area
	setAutoThreshold("Default"); // A threshold is used to remove the background and stay only
with the sphere
	run("Options...", "iterations=1 count=1");
	run("Convert to Mask"); // USe a mask to make the image binary
```

run("Watershed"); // separate possible areas interconnected and stay only with sphere

run("Analyze Particles...", "size=40000-Infinity display"); // Select areas that are upper a certain number of pixel. Thus the only possible area registered is the one of the sphere

close(); // close the image

close(); // CLose the summary and keep the results

}

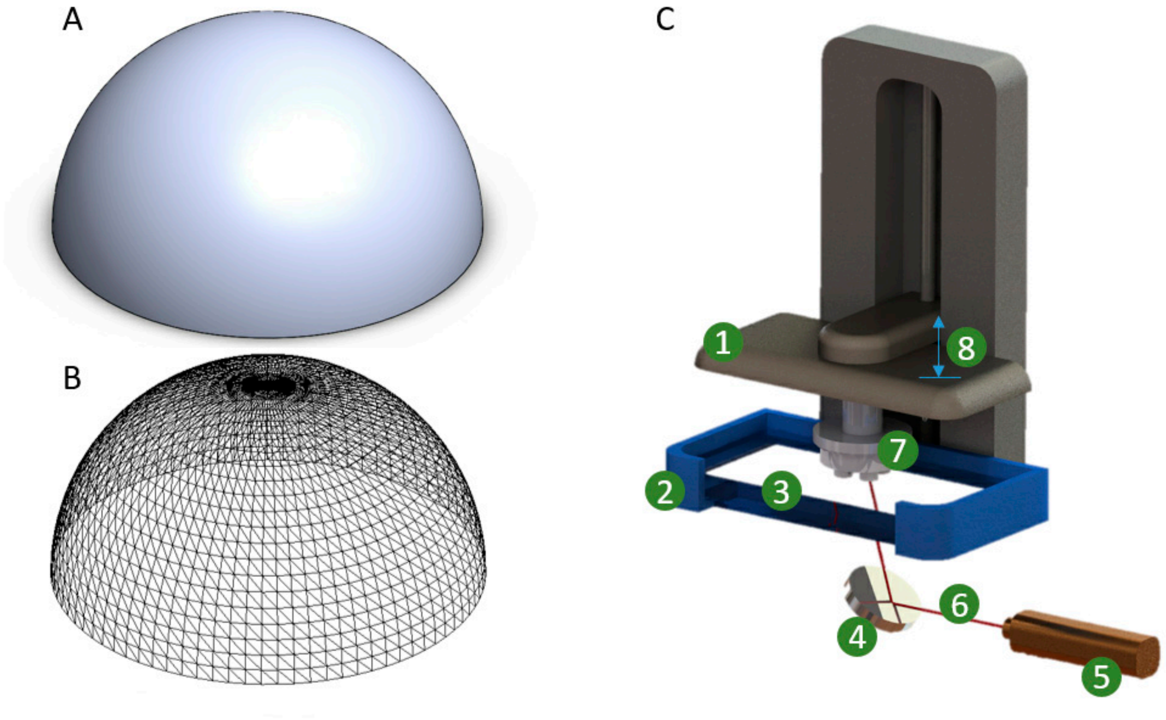

Supplementary Figure 1. 3D printing CAD, STL, and VP representation *A) A computer-aided design (CAD) model B) Standard triangle language (STL) file derived from the CAD model C) VP Printer where 1- is the building platform, 2- resin tank, 3- liquid resin, 4- XY scanning mirror, 5- UV laser source, 6 UV laser beam, 7- printed 3D structure, and 8- layer by layer elevation.*

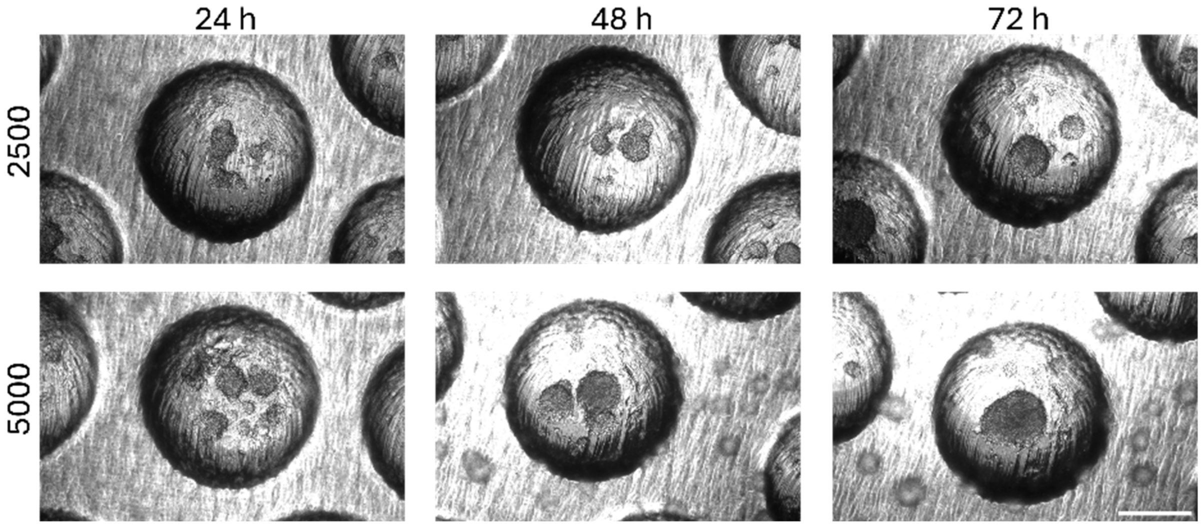

Supplementary Figure 2. U87 tumor spheroid formation in PDMS microwells without AARS. *Light microscopy of tumor spheroids with 2500 and 5000 cells/microwell at 24, 48, and 72 h after seeding, for molds without AARS treatment. Scalebar=500 µm.*

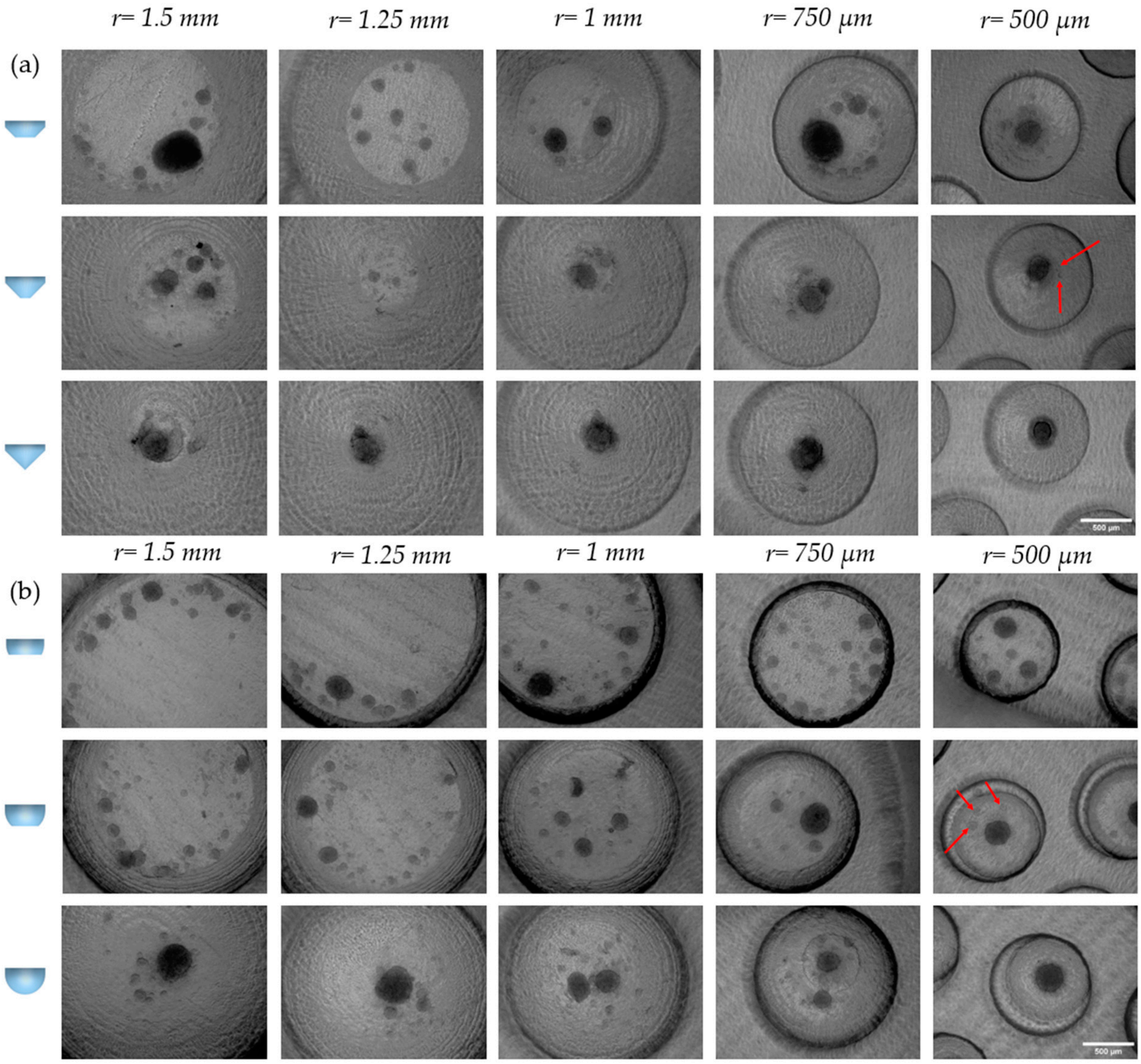

Supplementary Figure 3. EB formation in conical and semi-spherical microwells. *EBs formed in a) conical microwells truncated at 50%, 75%, and complete cone, and b) semi-spherical microwells truncated at 50%, 75%, and complete semi-sphere. Red arrows point to small cell aggregates or satellite spheroids. Scalebar = 500 µm.*

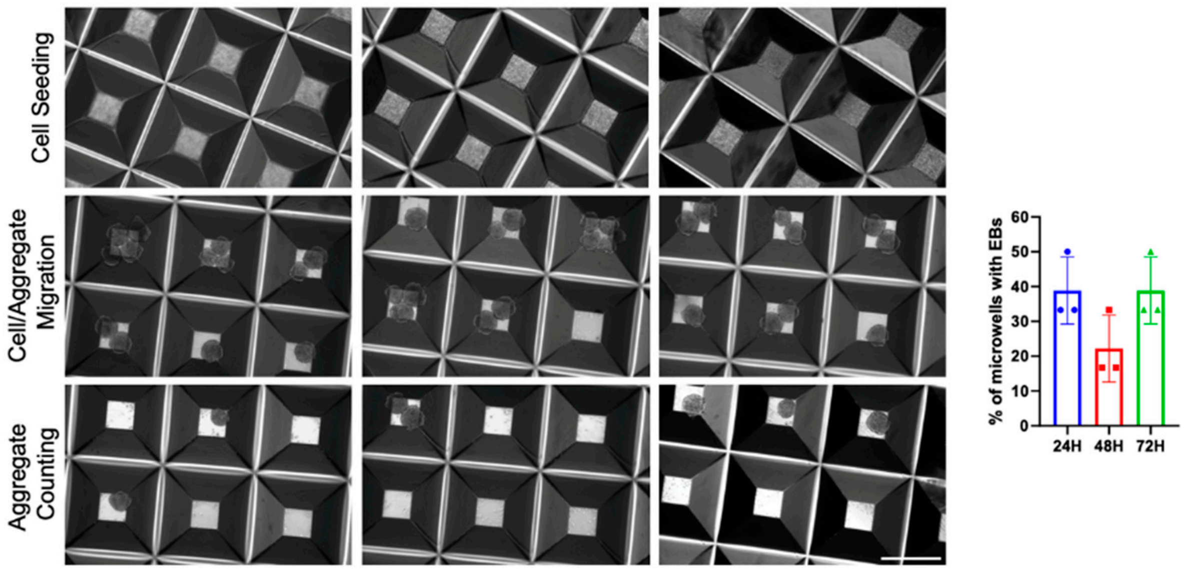

Supplementary Figure 4. Light microscopy of EBs on the Aggrewell 800 plates. *(upper row) Light microscopy images of Aggrewell 800 microwells immediately after cell seeding; (middle row) after 24h of incubation, showing empty wells and wells with multiple EBs, indicating cell/aggregate overflow; (lower row, from left to right) after 24h, 48h and 72h of incubation. Scalebar = 500 μm. Graph represent the percentages of single EBs per microwell at 24h, 48h and 72h after cell seeding (n=3).*
